# Disentangling Pantomime From Early Sign in a New Sign Language: Window Into Language Evolution Research

**DOI:** 10.3389/fpsyg.2021.640057

**Published:** 2021-04-14

**Authors:** Ana Mineiro, Inmaculada Concepción Báez-Montero, Mara Moita, Isabel Galhano-Rodrigues, Alexandre Castro-Caldas

**Affiliations:** ^1^Catholic University of Portugal, Lisbon, Portugal; ^2^Center of Interdisciplinary Research in Health, Catholic University of Portugal, Lisbon, Portugal; ^3^GRILLES, University of Vigo, Vigo, Spain; ^4^Linguistics Research Centre of the UNL (CLUNL), NOVA University of Lisbon, Lisbon, Portugal; ^5^University of Porto, Porto, Portugal; ^6^Centro de Linguística da Universidade do Porto, University of Porto, Porto, Portugal

**Keywords:** human communication, emergent sign language, pantomime, early signs, language evolution

## Abstract

In this study, we aim to disentangle pantomime from early signs in a newly-born sign language: Sao Tome and Principe Sign Language. Our results show that within 2 years of their first contact with one another, a community of 100 participants interacting everyday was able to build a shared language. The growth of linguistic systematicity, which included a decrease in use of pantomime, reduction of the amplitude of signs and an increase in articulation economy, showcases a learning, and social interaction process that constitutes a continuum and not a cut-off system. The human cognitive system is endowed with mechanisms for symbolization that allow the process of arbitrariness to unfold and the expansion of linguistic complexity. Our study helps to clarify the role of pantomime in a new sign language and how this role might be linked with language itself, showing implications for language evolution research.

## Introduction

A crucial turning point in human language evolution is the moment at which an individual is able to make references for things that are beyond what is immediately present. This ability to mimic traces of previous experiences and make absent things present is known as displacement. This is achieved by enacting, modeling or drawing a referent or some of its features by using the body (or parts of the body). This includes reference by concrete indexing—pointing with the index finger or with other body parts to some object, person or place associated with the absent item being referred to.

Certain properties of the communicative form (such as sign or spoken language phonology and co-speech gestures) and certain sensory-motor or affective properties of the corresponding referents might coincide (Fay et al., 2014; Perniss and Vigliocco, 2014). This is called iconicity.

Iconicity exists in any kind of sign that stands for a referent (Hookway, 2007). It appears in arts and literature, technology, and many other human products and practices, as well as obviously in both spoken and signed languages. In the former, the modalities that are part of the utterance, such as sounds and kinesic units, represent features of referents (in the case of metaphoric transference) or shared features of the referent which are associated to them (in the case of metonymy). Emblems (or quotable gestures) of spoken language (Kendon, 2004) are gestures that can be interpreted in the same way by the members of a linguistic community without the presence of speech. These are typical examples of the emergence and stabilization of form and meaning. The conventionalization of meaningful kinesic configurations generally occurs/develops as a response to communicative needs in a linguistic community. The conventionalization is motivated through a set of objects, actions or postures (which can be linked within each other) and idiomatisms (Payrató, 2014).

Iconicity occurs in communicative contexts, such as spontaneous iconic gestures and iconic conventionalized linguistic signs/words (Demey et al., 2008; Sandler, 2009; Taub, 2012). These two elements may be similar in the way they iconically represent the referent, but can differ both in the input structures and the resulting composition. In terms of the producer's intention, the iconic gesture illustrates the image of the referent and the iconic sign reveals the concept of the referent (Liddell, 2003; Cuxac and Sallandre, 2007). In addition, the iconic gesture occurs embedded in a specific discursive context making its interpretation impossible outside of its context. Instead, iconic signs are conventionalized and interpretable outside the discursive context, i.e., they are comprehensible when they occur in isolation. When it refers to the concept, the resemblance between iconic sign and concept is not always transparent, even when clearly iconic. Non-signers show difficulties in guessing the meaning of iconic signs (Klima and Bellugi, 1979; Pizzuto and Volterra, 2000), revealing that iconicity may motivate the sign formation but does not determine it. Research suggests that the iconic gesture evolves to a phonetic form that can no longer be freely changed (Aronoff et al., 2003). In this way, iconicity provides a key to understand language evolution, development and processing.

The nature of iconicity in sign language has been discussed with a focus on understanding whether this phenomenon is part of the formation of the sign (Taub, 2001, 2012) or the structuring principle of sign languages (Cuxac and Sallandre, 2007; Demey et al., 2008). In both spoken and signed languages, iconicity can be conveyed in all the modalities, however, the signs of sign language are in general more iconic than the coverbal gestures of speech, where iconicity can be simultaneously represented by vocal means (Perniss and Vigliocco, 2014).

The frequent and repeated imitation of a common action through body movements by the members of a group has been described as the origin for the stabilization and the conventionalization of simplified forms of body actions (Perniss and Vigliocco, 2014). Pantomime is defined/described as a whole-body process and can indeed engage body parts to represent objects and actions (Zywiczyński et al., 2018). Pantomime has been referred to as *enactment*, one of three techniques of representation, where “the gesturing body parts engage in a pattern of action that has features in common with some actual pattern of actions” (Kendon, 2004, p. 160). The other techniques, *modeling* and *depiction*, are a speciality of manual gestures, *referential manual actions* (to use Kendon's terminology). When observing the elements of sign languages, the classifiers may be considered the result of a depiction technique representation, since their function is to depict the size and shape, the location, the movement, and the handling of entities (depicted meanings) (Liddell, 2003).

In the context of deaf communication, in the case that there is no access and exposure to any conventional and structured linguistic system, pantomime represents a communication process outlined by the absence of language and relying on movements of the whole body or parts of the body. Pantomimes are action-oriented and involve the production of mimetic replications/reproductions of an action pattern (Sandler, 2009). Despite its non-linguistic character, pantomime is a unique semiotic resource for human communication because it allows a broad spectrum of meanings (Zywiczyński et al., 2018).

Within the research field studying the origins of language, we can consider two main streams of thought regarding the role of gestures and other visually perceived body movements (Corballis, 2003; Tomasello, 2008; Zlatev, 2008; Arbib, 2012) in the emergence of speech: the gesture-first (Hewes et al., 1973; Corballis, 2003) and the multimodal, or equal partners hypotheses (McNeill, 2012; Kendon, 2014). If we consider Corballis' and Kendon's points of view, they differ only with regard to the evolutionary timing and manner in which gesture was incorporated into language (Corballis, 2014). In fact, pantomime is compatible with multimodality and, regarding the origin of language, there is no need to assume that pantomime is unimodal (silent and visual). The pre-eminence of the visuomotor channel over the vocal auditory channel is triggered by iconic potential pantomime (Zywiczyński et al., 2018). Pantomimes are not easy to replicate and standardize because they emerge from creative processes and are interpreted on the spot. This is a powerful motivation for the conventionalization of pantomimic forms (Zywiczyński et al., 2018). In terms of the human-specific communicative system, pantomime can be characterized as dominated by gesture but also including vocalization, facial expression, and possibly the rudiments of depiction (Zlatev et al., 2020).

We will commence from the statement that gestures and vocalizations are intertwined from the very beginning of the language emergence in human beings. This point of view is supported by the following evidence: both modalities of language, oral and visuospatial, are closely linked: sign language involves not only manual articulation but also some vocalizations and movements of the face and hands, while spoken language is predominantly accompanied by manual co-speech gestures (Kendon, 1980, 1988; McNeill, 1992). Indeed, gesture research suggests that speech and gesture have the same underlying conceptual system (Kendon, 2004). In terms of human evolution, it makes sense that those systems (manual and vocal) co-evolved together and are intertwined with each other. Vocal and manual systems serve primary functions more critical to survival than language, and they must have been neurobiologically sophisticated from the beginning because they allowed man to take food to his mouth without biting his fingers (Mineiro, 2017, 2020).

Some studies suggest that the emergence of language could have been preceded by a stage of pantomimic communication (Corballis, 2003, 2009; Brown et al., 2019). Surprisingly, the production of pantomime and language relies on only partially distinct neural systems. A neuroimaging study revealed that pantomime production engages the superior parietal cortex bilaterally for deaf signers while sign language production (verbs in ASL) engages the left inferior frontal cortex. For hearing non-signers, pantomime production did not engage the left inferior parietal cortex (Emmorey et al., 2011). The authors of the study reported that the neural networks for pantomime generation are not identical for the deaf and hearing groups as deaf signers employ more extensive regions within the superior parietal cortex and hearing non-signers employ neural regions associated with episodic memory retrieval.

Some of these issues can be clarified from the homesigns literature and sign language acquisition data. When deprived from a conventional linguistic model as a sign language, deaf children create homesigns. They use both pointing gestures and iconic gestures to communicate. Their gestures convey particular meaning and based on their social situation, there is a need to create signs that are transparent and understandable, revealing an intrinsic segmental nature (Goldin-Meadow, 2005, 2012). Fuselier-Souza (2006) and Kendon (1980) have observed iconicity in homesigners and the stabilization or tendency to use the same form of these iconic signs in their lexicon in order to communicate efficiently. The striking need for communication in the human species leads, in the absence of a conventional linguistic system, to the creation of gestures that can be used as a shared baseline for conveying meaning.

Regarding pointing gestures, these also have an essential role in the language acquisition process in both hearing and deaf children. This is because they are grounded in joint attention, they facilitate interaction, and they trigger the child's entrance into a combinatory word system and, subsequently, acquisition of syntax. Morgenstern et al. (2010) found that monolingual deaf participants and bilingual hearing children produce pointing gestures earlier than monolingual hearing children. In the case of deaf monolingual participants, pointing gestures were used significantly more in comparison to others, and they soon combined with gestures or signs and get integrated into the linguistic system as a grammatical item.

In mature, established sign languages, pantomimic or gesture properties remain in grammaticalized signs as well as in pronominal signs that occur inherently with regard to a referential syntactic space (Cormier et al., 2013). Some core properties of grammatical nature and pointing gestures in pronoun signs are shared. This is particularly relevant as it reveals the principle of pantomimic nature in the formation of the sign.

Recent research using an artificial sign language in the lab showed that it takes fewer than five generations to pass from unconventional and straightforward pantomimes to stable iconic signs (Motamedi et al., 2016). These results highlight that learning and social interaction are crucial for the development of conventional signs and, subsequently, grammar, and support the pattern observed in the new Sao Tome and Principe Sign Language (Mineiro, 2017) and other emergent sign languages (Kegl et al., 1999).

In our study, the deaf group transitioned to conventionalized signs instead of pantomimes after a few months, and pantomimes evolved with modifications into stabilized and conventionalized signs. The social-cultural pressure stressed the evolutionary trajectory of this newly emergent language. In this paper, we will expose how quickly pantomimic communication turns into lexicalized and conventionalized signs in a newly emergent language. We will also argue that this process within learning and social interaction is a continuum and not a cut-off system. We adopt a broad concept of language (McNeill, 1992) to fully understand the role of pantomime and the role of a conventionalized sign which are intertwined with language itself.

## Materials and Methods

### Framework

Watching the birth of a language is a rare and fascinating privilege for any linguist, especially when in the context of promoting the structures that lead to the emergence of a language for those lacking it due to being deaf and living in isolation and linguistic deprivation. We had this privilege in Sao Tome and Principe. Sao Tome and Principe has a total of 187.000 inhabitants according to the last CENSUS in 2012. From this, 5,000 people (3% of the population) were born deaf or developed deafness due to various reasons, including medication for malaria during pregnancy (Caroça, 2017). Socio-economic development is low, and the nation lives mostly on external development aid (99%). Healthcare (primary care and specialities) is provided through development cooperation. It was during one such specialist mission that the otolaryngology team discovered the prevalence of deafness. Upon request, official authorization and with support of the Government of Sao Tome and Principe, we initiated a language intervention project in order to establish the foundations for an emerging sign language for deaf children to communicate and interact in, as these children were excluded from school due to their lack of ability to communicate.

### Project Design

In 2012–2013 the project “Without Barriers” arose, following the intention and necessity expressed by the Government of São Tome and Principe. This project was planned with the cooperation of the NGO *Instituto Marquês de Valle-Flôr, Cuf Infante Santo* Hospital and the Portuguese Catholic University and was co-funded by the Calouste Gulbenkian Foundation. This project aimed to promote and establish the foundations for the emergence of a sign language for the deaf, through an initial pilot group of 100 participants. The objective of this project was to encourage regular meetings (daily) among the deaf (children and juveniles) for a few hours per day to ensure that these children and juveniles got to socialize between themselves and create a common sign language together with the help of an experienced deaf instructor.

Considering the existing literature on emerging sign languages (Senghas et al., 1997; Meir et al., 2010), we decided to gather the deaf participants in a common place, similar to the case of Nicaraguan Sign Language (NSL), which seemed to be an appropriate solution for the Santomeans since they were geographically spread throughout two islands. Considering it was not possible to provide boarding for the deaf children and juveniles (as occurred in Nicaragua) due to the lack of logistical and financial means, we opted for finding a common place where the deaf participants could meet daily during the project which lasted for 2 years.

It was decided by the team not to “teach” any pre-existing sign language such as Portuguese Sign Language, the native language of the deaf instructor. This choice was based on the team's intention of not imposing any existing and structured language with a specific culture and history on the participants, but to afford the possibility of turning the participants into actors and thus builders of their own language. This non-imperialist linguistic policy led to the discovery of the “other deaf” as peers, and to the use of a common communication system among them to contribute to the linguistic *boom* that was observed along the 2-year project duration. The social interaction between the deaf participants led to the conventionalization and stabilization of the linguistic forms (Mineiro, 2017).

### Participants

Given the logistical, financial, and time constraints of the project, it was impossible to include all the Santomean deaf, who numbered around 5,000 individuals presenting various degrees of deafness (light, moderate, severe, and profound). Thus, the team chose to work with a pilot group covering only youngsters that were profoundly deaf. The participants in this pilot group were chosen based on a survey assessing deafness degree conducted by the otorhinolaryngology team from CUF Infante Santo Hospital and on the data provided by the Sao Tome and Principe Government (Ministry of Education, Culture and Science). Therefore, a group of children and young individuals with profound deafness, who did not attend school because their teachers could not teach them owing to their deafness and the lack of a common language, were chosen as participants.

Non-verbal cognitive tests (Raven's Progressive Matrices and the Human Figure test) were applied to the selected group by experienced psychologists to understand whether the participants were only deaf, or whether they presented other cognitive co-morbidities which inhibited natural language acquisition. All subjects in the tested sample revealed an average or above-average non-verbal intelligence. A total of 100 participants were recruited for the pilot study. Participants were aged between 4 and 25, 68% of deaf participants were minors (from 4.06 to 17.07 years old) and 32% of deaf participants were adults (from 18.01 to 25.02 years old). The gender balance in the sample was 80 deaf female individuals and 20 deaf male individuals. All participants lacked reading and writing skills as they did not attend school due to the reasons mentioned above. The sample was a convenience sample.

Sociolinguistic data were collected through oral questionnaires with fixed questions (e.g., age, schooling, number of people living in the same house, the language used) conducted with the families of the participants and semi-structured interviews allowing the interviewee to express the feelings and difficulties about having a deaf relative and also to report the kind of communication used with this relative.

All participants belonged to large hearing families (with four or more siblings per family), and few of them (only two participants) had deaf siblings who communicate between them in signed home communication. The families of the participants were extended, including not only the father and mother nucleus (many of them only had the biological mother) but also grandparents, uncles, cousins and half-brothers.

As for the linguistic environment of the deaf participants' families, it was characterized by the use of Forro (the most frequently used creole of Sao Tome and Principe) at home, and by the use of Portuguese at school (siblings of the participants and interactions between family and school). Assuming that isolated deaf children tend to develop homesigns sytem and that when these children are brought together their homesign systems seem to adjust to each other (Goldin-Meadow, 2005, 2012), we developed a questionnaire, a semi-structured interview and made regular visits to their family context to find if there was this kind homesign system. The conditions of socialization of deaf people in their families were also observed in addition to the linguistic environment, first through the same questionnaire and semi-structured interview, and then through specific but regular visits to the families over 2 years, during the time period of the project. Hence, we found that the deaf family members in the pilot group did not maintain much communicative interaction with the other family members. These children were not well-integrated in their families (Mineiro and Carmo, 2016). In the case of deaf participants with deaf siblings, they developed a gestural communication characterized by homesigns with each other but not much within the rest of the family. We observed (Mineiro and Carmo, 2016) that the communication within the deaf siblings' families had specific characteristics, such as short vocabulary, no syntactic rules and variation in the gestures. The siblings' communication was simplified, as it was constructed impromptu, or by convention, between them. Thus, it may be framed within a pidgin (Bickerton, 1981).

In the remaining cases, the interaction was conducted through pointing gestures accompanied by vocal sounds, mimics and coded-gestures, which were created within the hearing family members to meet daily communication needs. In these participants, we observed a different form of communication from the one observed in deaf siblings. The characteristics of these gestures were based on pointing accompanied by vocal sounds and pantomimes. These gestures were mostly non-systematic and non-repeated gestures. We also found some agreed-upon gestures (coded-gestures) to communicate basic needs with their hearing family members, for example, a coded-gesture for PAIN (facial expression of pain + pointing to the painful part of the body) or a coded-gesture for HUNGER (pointing to mouth + vocal sound).

In our view, the boundary between homesigns produced by the deaf siblings and the common communication used by the other deaf participants in our study might be considered tenuous. However, following the literature, we consider that homesigns are created by deaf children and are stable in form (Goldin-Meadow, 2012; Shneidman and Goldin-Meadow, 2012). As for pantomime gestures, they are iconic gestures engaging a whole-body process or body parts in isolation (Zywiczyński et al., 2018). We distinguished coded-gestures from homesigns as these are created within the hearing family members to meet daily communication needs. Both pantomime and coded-gestures do not reveal any stabilization of form as homesigns do.

It should also be noted that the deaf participants in our project did not know each other prior to meeting through the project. These children and youngsters lived in an environment of social isolation and linguistic deprivation because they did not live among deaf people and lacked deaf association.

It is essential to highlight that social interaction is crucial for language to exist. The fact that humans are biologically programmed for a language does not mean that they acquire a language or “activate” language proficiency if they do not have contact with other human beings who share the same linguistic modality (oral or sign). Homesigns are an agreed-upon type of communication to conduct fundamental interactions but that do not lead to language development (Goldin-Meadow, 2012). Only various and frequent social interactions between the users of the same linguistic modality can result in the development of a language whose structural complexity grows in time (Meir et al., 2010).

### Procedure

The project *Without Barriers*, with the above-described pilot group, took place between February 2013 and February 2015. Several goals were set out during the 2-year duration of the project, which were (i) Final selection of the sample of participants (ii) Daily language contact sessions over the 2 years of the project (with 2-week breaks at Christmas and Easter and 1-month break during the summer) (iii) weekly sessions of joint activities (market trips, beach, walks); (iv) elaboration of the first collection of gestural vocabulary to be drafted and disseminated (Carmo et al., 2014). Afterwards, 30% of the collected linguistic data were analyzed, outside the project's time limits (2013–2018).

The necessary means of transportation were provided through the funding obtained for the project, so that the geographically dispersed participants could participate daily in the project activities. Full-time availability of a deaf instructor with a Master's degree and with Portuguese Sign Language as a native language, who was experienced in teaching the deaf, was also ensured. Two rooms were provided for running the daily sessions.

The work carried out was closely followed and monitored by the Ministry of Education, Culture and Science as well as by the other partners. The project funding also covered all the school and session materials as well as the production of first dictionary edition of Sao Tome and Principe's Sign Language (Carmo et al., 2014).

The younger participants met in the designated room in the village of Santana every day for the scheduled sessions in the mornings and numbered about 25 participants per session. There were two morning sessions, the first beginning at 8 a.m. and the second beginning at 10.30 a.m. Each session lasted 2 h. The age of participants attending these sessions ranged between 4 and 14. In the afternoon (3–5 p.m.), a session for the older 50 participants was provided (15–25 years old) in the village of Bombom. Age criteria (peer identification) and logistic conditions (impossibility of having 100 participants in a single session) led to the organization of these two groups. However, the participants of Santana would go to Bombom in the afternoon for extracurricular activities like drawing and theater, and those in Bombom would go to Santana in the morning for the same purpose. These two villages are geographically close (10 km) on Sao Tome island. In this way, the recreation places were common, and thus there was the opportunity for contact and interaction between the deaf participants of different ages included in the project.

From 2013 to 2015, various activities were organized at weekends (Saturdays and Sundays), where younger and older deaf individuals would meet outside the sessions, such as going to the market, washing clothes in the river, going to the beach, or having a walk together. In these outside sessions, learning and transmission between the participants were encouraged, although it was not possible to keep a videographic registration. These meetings were coordinated by the deaf instructor, who followed most of these activities weekly. All of the outside meetings were reported on the following Monday, either in the morning during the session in Santana, or during the session in Bombom in the afternoon, and these were always recorded.

The corpus was collected based on an elicited task. The deaf instructor used picture cards to induce gestural productions that should correspond to the presented referents. Hence, deaf participants had to suggest signs and discuss in groups the more adequate proposal. In this task, the deaf participants were free to share experience and knowledge about the referent. Some of these proposed signs were brought from communication at home (homesigns) yet they would be later modified by the deaf group with common signs that were usually different from homesigns (Goldin-Meadow, 2012). Interestingly, the deaf siblings with a homesign communication system were more active in proposing gestures and they were followed by some of the shyer classmates. Sometimes, the shy classmates simply imitated the gesture previously given; at other times they provided other different gestures with similarities with the first sign given (Mineiro et al., 2017). The instructor did not use Portuguese Sign Language, but when she needed to interact with the participants, she resorted to pantomime.

**Figure 1:**
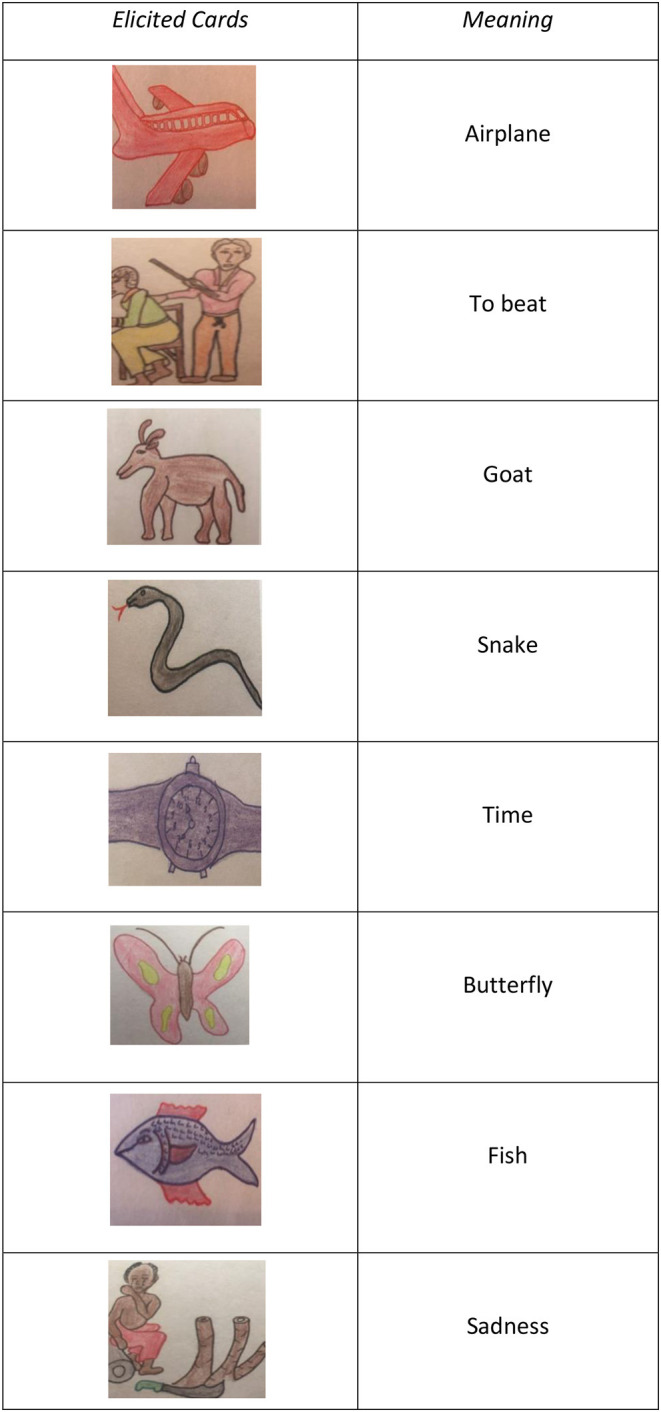
Draws presented to the participants in the elicitation task.

In order to elicit signs, the instructor used a series of 100 picture cards that were designed by local artists in such a way that the participants could recognize the cultural representations on the cards (see Figure 1). The images depicted on the cards included objects (e.g., glass, bed, food, means of transportation, money), emotions (e.g., happy, sad, angry, etc.), and images that tell stories (e.g., going fishing, shopping at a supermarket, scenes from the household or everyday life). Only cards with simple images (e.g., objects) were used during the first stage (first 6 months), and in the second stage (after 6 months), the story images were employed.

All in-room sessions were recorded with two cameras systematically placed in such a way that they would cover two deaf interlocutors. Later, these recordings were placed in a database accommodating this corpus at the Catholic University of Portugal, and then (between 2015 and 2018) 20% of these videos were transcribed for gloss and for literal translation through the Eudico Language Annotator program, hereafter ELAN. Twenty percentage of the transcription was made by the deaf instructor who had collected the data during the sessions. The revision stage of the transcribed data was performed by a second deaf signer who learned Sao Tome e Principe Sign Language during his stay on the island of Sao Tome. Both transcribers have experience in transcription of sign data. Concordance between more than 80% of the transcribed items was obtained, resulting in an interjudge agreement (Fortin, 2009). The few disagreements were solved by a third sign transcriber who had contact with the sign Sao Tome e Principe Sign Language at different times during the project.

Although other videos have only been transcribed at a gloss level, they have all been analyzed, and the necessary information such as the frequency of signs and the evolution of pantomimic gestures to lexicalized signs have been collected. These analyzed videos correspond to 5% of the total corpus.

Thus, in this study, we analyzed 110 video recordings of 60 min each, which constitute 25% of the total corpus. This sample is representative of the corpus which was collected in four stages: videos from the early phase of the project (February to July 2013), videos from the intermediate phase of the project (September 2013 to February 2014), videos from the pre-final phase (March 2014 to July 2014) and videos from the final phase of the project (September 2014 to February 2015). As it was a 2-year project, and in order to see the language evolution in this scope of time, we decided to divide the sample corpus into 4 stages with 6 months' time difference between each phase.

We observed 1,000 items which included signs and pantomimic gestures produced in the elicited context, and over the four stages of data collection, only 759 were established signs (signs or classifiers) of a newly-born sign language. These 759 signs occur systematically in our corpus in the fourth phase of data collection representing Level 3 and 4.

The levels of frequency were based on the total frequency of occurrences in the elicited corpus. The criterion of the levels of frequency distribution is based on the data-driven corpus analysis (Gierut and Dale, 2007; Gries, 2008).

At level 1 and 2, we identified gestures that were produced <25% of the time in the corpus. These were considered low frequency gestures, which means that a given gesture occurred less in than 25% of the responses to an elicited-card. Thus, these gestures were not analyzed and added into the dictionary of fundamental vocabulary of Sao Tome and Principe Sign Language (Carmo et al., 2014).

At level 3 and 4, we identified gestures that occurred more than 26% of the time in the corpus. These were considered high frequency gestures, which means that a given gesture occurred in more than 26% of the responses to an elicited-card. The gestures in both levels 3 and 4 were analyzed and added to the dictionary of fundamental vocabulary (Carmo et al., 2014). This is all clarified in the list below:

1. Level 1: Sign or gesture that occurs in 1–5% of the analyzed corpus
2. Level 2: Sign or gesture that occurs in 16–25% of the analyzed corpus
3. Level 3: Sign or gesture that occurs in 26–50% of the analyzed corpus
4. Level 4: Sign or gesture that occurs in 51–100% of the analyzed corpus

## Results

To distinguish between pantomimic gestures and signs of a new language, we used the characteristics previously described in the study of early signs in Sao Tome and Principe Sign Language (Mineiro et al., 2017). Pantomime was defined as an iconic gesture that shows a strong resemblance with its referent (Perniss and Vigliocco, 2014), a whole-body process or engagement of isolated body parts to represent objects and their actions (Sandler, 2009; Zywiczyński et al., 2018). The first signs in Sao Tome and Principe Sign Language were described as having trends of emergent phonology and morphology as well as combinatory and recursive characteristics revealed in the produced sentences. The earlier signs in Sao Tome and Principe Sign Language also showed the critical role of iconicity in their formation (Mineiro et al., 2017).

### Pantomimic Gestures and Signs in Numbers and Graphs

In the first analysis, we have distinguished the pantomimic gestures from signs, classifiers and other gestures. The critical division between other gestures and sign is the result of a deliberate decision to exclude conventional co-sign gestures (Müller, 2018). As for classifiers, they were not considered pantomimic gestures given they may have inherent grammatical features and they universally use a system that depicts the movement and location of objects in space (e.g., Liddell, 2003), showing a possible development from a depiction strategy which differs from pantomime nature (Kendon, 2004; Sandler, 2009).

**Graph 1:**
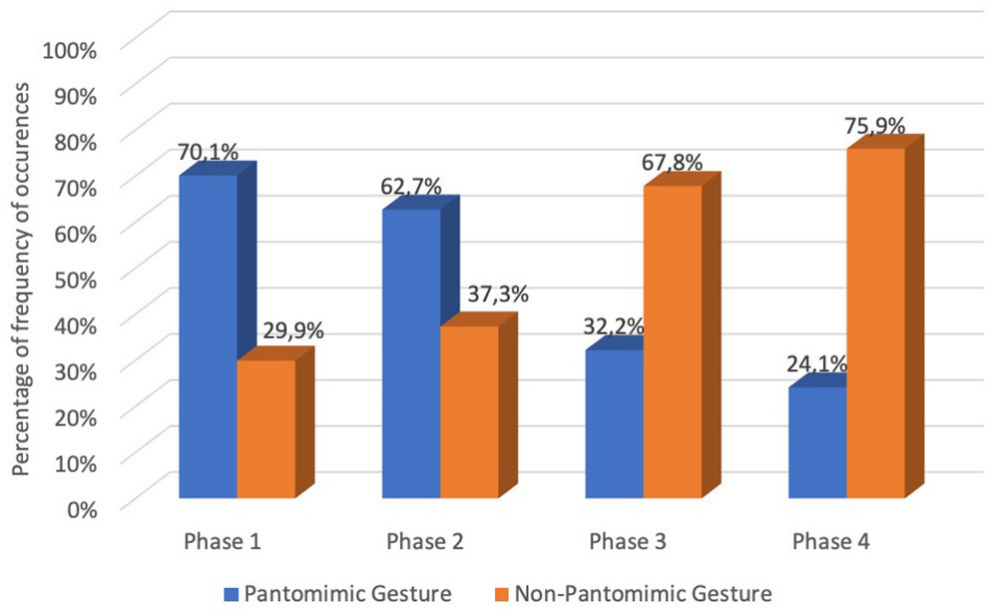
Percentage of frequency of occurrences of pantomime gestures and sign, classifiers, and other gestures along the four phases of collecting data.

In the early phase (phase 1) of our data collection, we found 70.1% of our data consisted of pantomimic gestures, and 29.9% consisted of signs, classifiers and other gestures. 62.7% of the intermediate phase data comprised pantomimic gestures, and 37.3% comprised signs, classifiers and other gestures. In the pre-final phase (phase 3), 32.2% of analyzed occurrences consisted of pantomimic gestures and 67.8% consisted of signs, classifiers and other gestures. 24.1% of the final phase data consisted of pantomimic gestures, and 75.9% consisted of signs, classifiers and other gestures. Thus, looking at Graph 1, it appears that pantomime predominates in the first two phases [phase 1 (70.1%) and phase 2 (62.7%)], whereas in phases 3 and 4, we may observe that sign, classifiers and other gestures are predominant [phase 3 (67.8%) and phase 4 (75.9%)].

In order to find differences in the prevalence of pantomime gestures between each pair of phases, we applied the McNemar test. This non-parametric test allows the comparison of counts in 2 × 2 tables and is indicated for use in dichotomous scales when comparing two repeated samples (in this case two evolution phases).

The McNemar test revealed a significant difference (for *p* < 0.001) in the number of pantomime gestures occurrences between all phases (Table 1). From phase 1 to phase 2, there was a reduction of 7.4% in the occurrence of pantomime gestures. From phase 2 to phase 3, there was a reduction of 30.5% in the occurrence of pantomime gestures. From phase 3 to phase 4, there was a reduction of 8.1% in the occurrence of pantomime gestures. Pantomime decreased significantly throughout the analyzed phases.

**Table 1:** McNemar test: Comparison of pair of phases based on the number of occurrences of pantomimic gestures and non-pantomimic gestures in the analyzed 1,000 gestures and signs.

	**Phase 1 vs. Phase 2**	**Phase 2 vs. Phase 3**	**Phase 3 vs. Phase 4**	**Phase 1 vs. Phase 3**	**Phase 1 vs. Phase 4**	**Phase 2 vs. Phase 4**
**PG/PG** Nb of PG that occurred in both phases	62.7% (627)	32.2% (322)	24.1% 241	37.9% (379)	46.0% (460)	24.1% (241)
**NPG/NPG** Nb of NPG that occurred in both phases	29.9% (299)	37.3% (373)	67.8% (678)	29.9% (299)	29.9% (299)	37.3% (373)
**PG/NPG** Nb of PG that become NPG from one phase to the next	7.4% (74)	30.5% (305)	8.1% (81)	32.2% (322)	24.1% (241)	38.6% (386)
**NPG/PG** Nb of NPG that become PG from one phase to the next	0% (0)	0% (0)	0% (0)	0% (0)	0% (0)	0% (0)
Total nb of gestures and signs	1,000	1,000	1,000	1,000	1,000	1,000
Test McNemar	*p < *0.001	*p < *0.001	*p < *0.001	*p < *0.001	*p < *0.001	*p < *0.001

*PG, pantomimic gesture; NPG, non-pantomimic gesture*.

**Figure 2:**
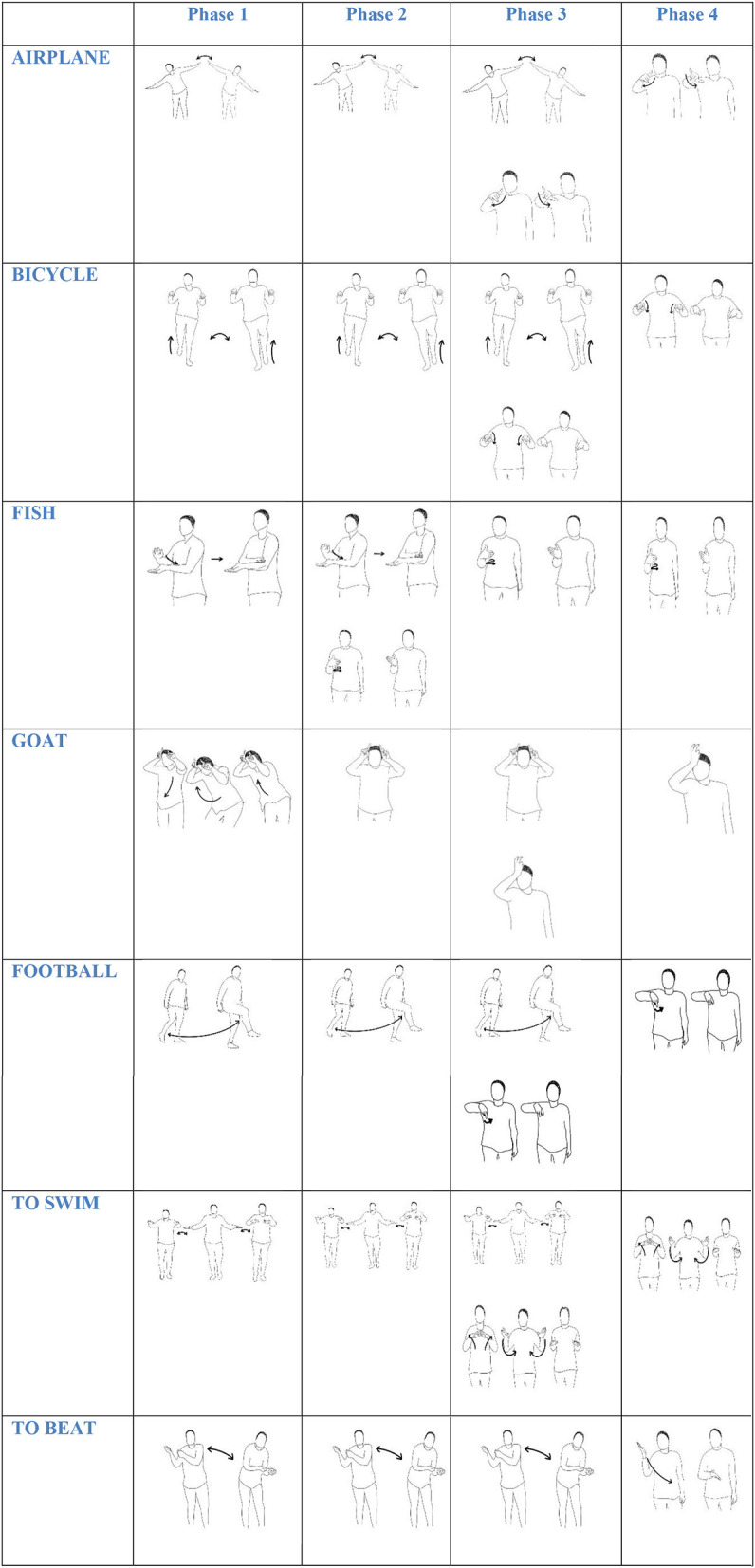
Pantomimic gestures and signs across the four phases.

The stress to communicate among this population led to mechanisms through which systematicity arose. The cultural pressure for everyday communication triggered the language to adapt itself to use and transmission. Over 2 years of linguistic immersion, pantomime significantly decreased, with a low frequency of occurrence in the last analyzed phase (24.1%), and signs and classifiers significantly increased, as the following examples show. Signs like PLANE, BICYCLE, FISH, GOAT, FOOTBALL, TO BEAT, and TO SWIM were first pantomimic gestures that evolved to lexicalized signs as seen in Figure 2.

### Step by Step: From Pantomime to Sign

Constructing complexity in a newly-born sign language is accompanied by the development of linguistic signals specialized for particular linguistic functions (Dachkovsky et al., 2018) and by the reduction of articulatory effort (Lehman, 2008; Bybee, 2010; Dachkovsky et al., 2018). This reduction is due to the decrease of biomechanical forces involved in the articulation. As Kirchner (1998, 2004) stated, spoken languages have a well-known drive for ease of articulation which can extend to sign languages (Napoli et al., 2014). Change comes gradually, and old forms slowly die, living together for some time with new forms within variation and oscillation.

Some of the forms expressed in Figure 2, especially in phases 2 and 3 (e.g., AIRPLANE, FISH, GOAT, TO SWIM), show this process of change over time specifically, gradually keeping the balance between comprehension and economy (Dachkovsky et al., 2018). In terms of economy and ease of production, pantomimic gestures meaning AIRPLANE or GOAT or SWIM, or BEAT or FOOTBALL became more comfortable to produce when they turned into conventionalized signs, losing the involvement of body parts such as the torso, legs and head. This body parts were analyzed as articulators involved in the 1,000 gestures and signs.

**Graph 2:**
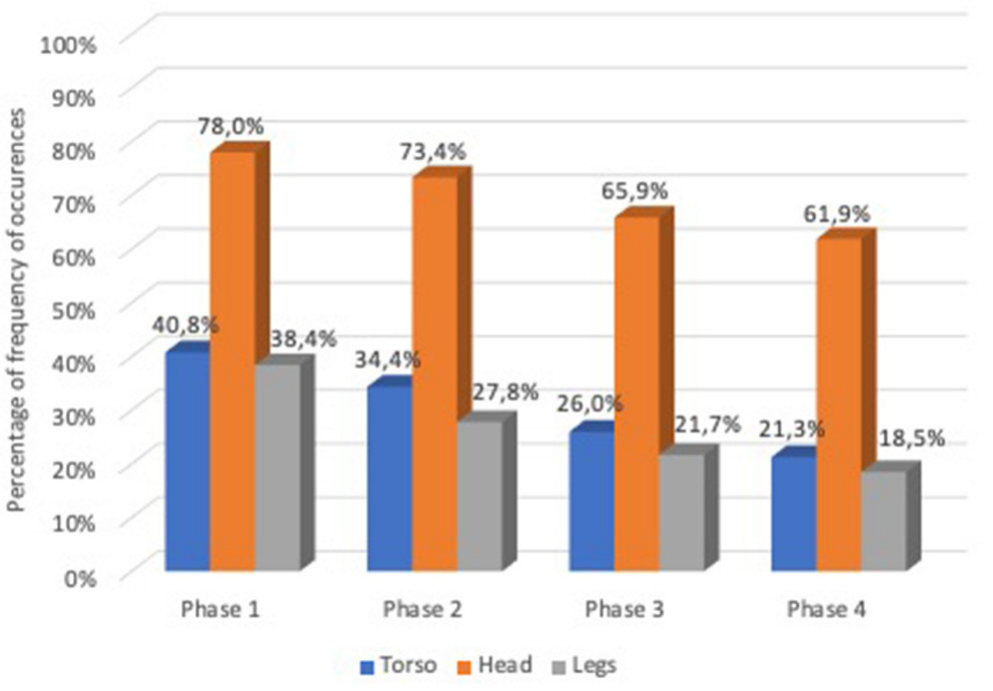
Percentage of frequency of occurrences of articulators use torso, head and legs in the analyzed 1,000 gestures, and signs.

Regarding the frequency of occurrences of these three body parts in the analyzed 1,000 gestures and signs in the corpus, it was observed that in phase 1, use of the torso occurred in 40.8% of the data, the use of the head occurred in 78% of the data and use of the legs occurred in 38.4% of the data. In phase 2, use of the torso occurred in 34.4% of the data, use of the head occurred in 73.4% of the data and use of the legs occurred in 27.8% of the data. In phase 3, use of the torso occurred in 26% of the data, use of the head occurred in 65.9% of the data and use of the legs occurred in 21.8% of the data. In phase 4, use of the torso occurred in 21.3% of the data, use of the head occurred in 61.9% of the data, and use of the legs occurred (in) 18.5% of the data in the total of 1,000 items. In all phases, the head was revealed to be the most present articulator in the total of analyzed gestures and items in the data (Graph 2).

In order to verify the existence of significant differences between each pair of phases in the number of occurrences of use of the articulators torso, head and legs, the McNemar test was used.

The McNemar test revealed a significant difference (*p* < 0.0001) between all phases in the reduction of number of occurrences of torso use as an articulator in the 1,000 gestures and signs. From phase 1 to phase 2, there was a reduction of 6.4% of occurrences (Table 2). From phase 2 to phase 3, the reduction of occurrences was 8.1%. And from phase 3 to phase 4, the reduction of occurrences was 4.7%. The statistical analysis shows us that the use of the torso as an articulator in the gesture and sign articulation decreased along the phases.

**Table 2:** McNemar Test: Comparison of pair of phases based on the number of gestures or signs in which torso occurred and not occurred considering the analyzed 1,000 gestures and signs.

**Trunk**	**Phase 1 vs. Phase 2**	**Phase 2 vs. Phase 3**	**Phase 3 vs. Phase 4**	**Phase 1 vs. Phase 3**	**Phase 1 vs. Phase 4**	**Phase 2 vs. Phase 4**
**Torso/Torso** Nb of gestures or signs that torso occurred in both phases	34.4% (344)	26.0% (260)	21.3% (213)	26.0% (260)	21.3% (213)	21.3% 213
**No-Torso/No-Torso** Nb of gestures or signs that had no occurrence of torso in both phases	59.2% (592)	65.6% (656)	74.0% (740)	59.2% (592)	59.2% (592)	65.6% (656)
**Torso/No-Torso** Nb of gestures or signs where torso disappeared from one phase to the next	6.4% (64)	8.1% (81)	4.7% (47)	14.8% (148)	19.5% (195)	13.1% (131)
**No-Torso/Torso** Nb of gestures or signs that torso was added from one phase to the next	0% (0)	0% (0)	0% (0)	0% (0)	0% (0)	0% (0)
Total nb of gestures and signs	0% (0)	0% (0)	0% (0)	0% (0)	0% (0)	0% (0)
Test McNemar	*p < *0.001	*p < *0.001	*p < *0.001	*p < *0.001	*p < *0.001	*p < *0.001

For the statistical analysis on the occurrence of use of the head as an articulator, the McNemar test reveals a significant difference (*p* < 0.001) between all phases in the reduction of number of occurrences of head use as an articulator in the 1,000 gestures and signs (Table 3). From phase 1 to phase 2, there was a 4.6% reduction of head articulator occurrences. From phase 2 to phase 3, the reduction was 7.5%, and from phase 3 to phase 4, the reduction was 4.0% of head use occurrences. The statistical analysis shows us that use of the head as an articulator in the gesture and sign articulation decreased along the four phases.

**Table 3:** McNemar Test: Comparison of pair of phases based on the number of gestures or signs in which head occurred and not occurred considering the analyzed 1,000 gestures and signs.

**Head**	**Phase 1 vs. Phase 2**	**Phase 2 vs. Phase 3**	**Phase 3 vs. Phase 4**	**Phase 1 vs. Phase 3**	**Phase 1 vs. Phase 4**	**Phase 2 vs. Phase 4**
**Head/Head** Nb of gestures or signs that head occurred in both phases	73.4% (734)	65.9% (659)	61.9% (619)	65.9% (659)	61.9% (619)	61.9% (619)
**No-head/No-head** Nb of gestures or signs that had no occurrence of head in both phases	22.0% (220)	26.6% (266)	34.1% (341)	22.0% (220)	22.0% (220)	26.6% (266)
**Head/No-head** Nb of gestures or signs where head disappeared from one phase to the next	4.6% (46)	7.5% (75)	4.0% (40)	12.1% (121)	16.1% (161)	11.5% (115)
**No-head/Head** Nb of gestures or signs that head was added from one phase to the next	0% (0)	0% (0)	0% (0)	0% (0)	0% (0)	0% (0)
Total nb of gestures and signs	100% 1,000	100% 1,000	100% 1,000	100% 1,000	100% 1,000	100% 1,000
Test McNemar	*p < *0.001	*p < *0.001	*p < *0.001	*p < *0.001	*p < *0.001	*p < *0.001

For the statistical analysis on the occurrence of using the legs as an articulator, the McNemar test revealed a significant difference (*p* < 0.001) between all phases in the reduction of number of occurrences of leg use as an articulator in the 1,000 gestures and signs (Table 4). From phase 1 to phase 2, there was a reduction of occurrences of 10.6%. From phase 2 to phase 3, there was a 6.1% reduction in these gestures and signs. And from phase 3 to phase 4, the reduction of leg use was 3.2%. The statistical analysis shows us that use of the legs as an articulator in the gesture and sign articulation decreased along the four phases.

**Table 4:** McNemar Test: Comparison of pair of phases based on the number of gestures or signs in which legs occurred and not occurred considering the analyzed 1,000 gestures and signs.

**Legs**	**Phase 1 vs. Phase 2**	**Phase 2 vs. Phase 3**	**Phase 3 vs. Phase 4**	**Phase 1 vs. Phase 3**	**Phase 1 vs. Phase 4**	**Phase 2 vs. Phase 4**
**Legs/Legs** Nb of gestures or signs that legs occurred in both phases	27.8% (278)	21.7% (217)	18.5% (185)	21.7% 217	18.5% (185)	18.5% (185)
**No-legs/No-legs** Nb of gestures or signs that had no occurrence of legs in both phases	61.6% (616)	72.2% (722)	78.3% (783)	61.6% (616)	61.6% (616)	72.2% (722)
**Legs/No-legs** Nb of gestures or signs where legs disappeared from one phase to the next	10.6% (106)	6.1% (61)	3.2% (32)	16.7% (167)	19.9% (199)	9.3% (93)
**No-legs/No-legs** Nb of gestures or signs that legs were added from one phase to the next	0% (0)	0% (0)	0% (0)	0% (0)	0% (0)	0% (0)
Total nb of gestures and signs	100% 1,000	100% 1,000	100% 1,000	100% 1,000	100% 1,000	100% 1,000
Test McNemar	*p < *0.001	*p < *0.001	*p < *0.001	*p < *0.001	*p < *0.001	*p < *0.001

**Graph 3:**
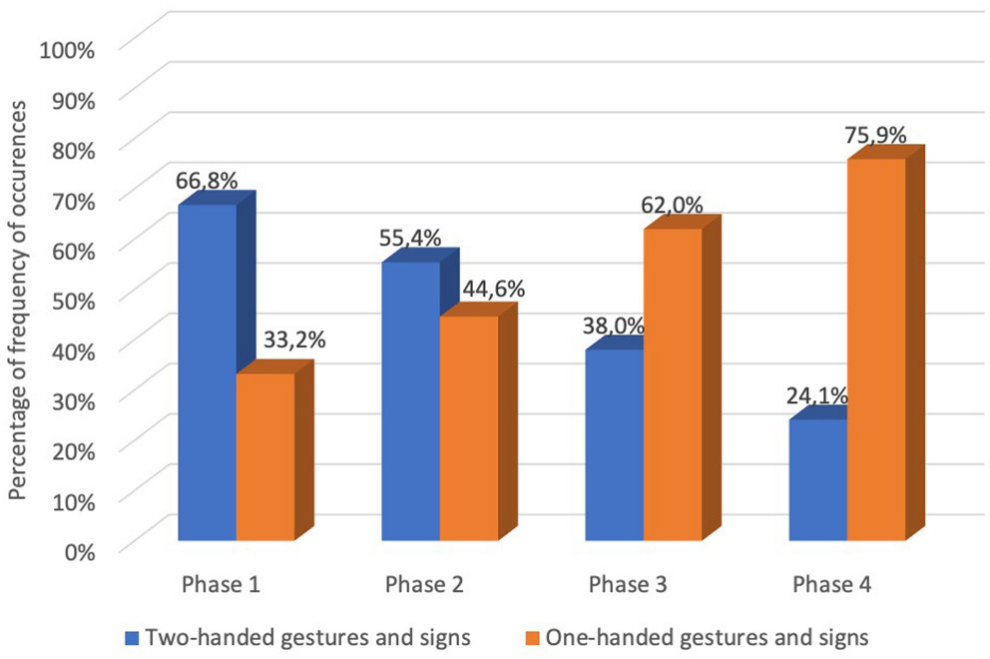
Percentage of frequency of occurrences of one hand and two hands articulators in the analyzed 1,000 gestures and signs along the four phases.

Observing that the evolution from pantomimic gestures to sign tended to reduce the use of articulators, we analyzed the occurrences of one and two hand use as articulators in the analyzed 1,000 gestures and signs along the four phases. In phase 1, two hands occurred in 66.8% and one hand occurred in 33.2% of gestures and signs. In phase 2, two hands occurred in 55.4%, and one hand occurred in 44.6%, of gestures and signs. In phase 3, two hands occurred in 38%, and one hand occurred in 62.0% of gestures and signs. In phase 4, the two hands occurred in 24.1%, and one hand occurred in 75.9% of gestures and signs (Graph 3). The analyzed data revealed a decrease in two-handed gestures and signs (for example, see GOAT, AIRPLANE, FOOTBALL, Figure 2).

To find differences in the occurrence of one-hand and two-hand articulators between the four phases in the 1,000 gestures and signs, we applied McNemar test to the data. This test revealed a significant difference (*p* < 0.001) between all phases in the number of occurrences of two-handed and one-handed gestures and signs (Table 5). From phase 1 to phase 2, there was a reduction of 11.4% in the number of occurrences of two-handed gestures and signs. From phase 2 to phase 3, the reduction was 17.4% of number of occurrences of two-handed gestures and signs. From phase 3 to phase 4, the reduction was 13.9% of number of occurrences of two-handed gestures and signs.

**Table 5:** McNemar Test: Comparison of pair of phases based on the number of one-handed and two-handed gestures or signs occurrences considering the analyzed 1,000 gestures and signs.

	**Phase 1 vs. Phase 2**	**Phase 2 vs. Phase 3**	**Phase 3 vs. Phase 4**	**Phase 1 vs. Phase 3**	**Phase 1 vs. Phase 4**	**Phase 2 vs. Phase 4**
**Two-handed/Two-handed** Nb of two-handed gestures and signs in both phases	55.4% (554)	38.0% (380)	24.1% (241)	38.0% (380)	24.1% (241)	24.1% (241)
**One-handed/One-handed** Nb of one-handed gestures and signs in both phases	33.2% (332)	44.6% (446)	62.0% (620)	33.2% (332)	33.2% (332)	44.6% (446)
**Two-handed/One-handed** Nb of gestures or signs that turned to one-handed from one phase to the next	11.4% (114)	17.4% (174)	13.9% (139)	28.8% (288)	42.7% (427)	31.3% (313)
**One-handed/Two-handed** Nb of gestures or signs that turned to two-handed from one phase to the next	0% (0)	0% (0)	0% (0)	0% (0)	0% (0)	0% (0)
Total nb of gestures and signs	100% 1,000	100% 1,000	100% 1,000	100% 1,000	100% 1,000	100% 1,000
Test McNemar	*p < *0.001	*p < *0.001	*p < *0.001	*p < *0.001	*p < *0.001	*p < *0.001

As previously studied by Dachkovsky et al. (2018), linguistic complexity is grounded in (i) reduction of articulatory effort as well as a reduction in size of articulators. Regarding amplitude (the articulation in the signing space) it is eventually not a question of linguistic complexity but more an economical mechanism of languages, although, this economical mechanism only occurs during the conventionalization process.

To measure the amplitude of a sign or a gesture [signing space (Nyst, 2007)], we used a dichotomic measure, that is extended *signing* (amplitude –) vs. *compact signing* (amplitude +). Extended signing is done with the arms extended from the center of the body (e.g., see the sign AIRPLANE, phase 1, 2, 3 Figure 2). Compact signing is accomplished when the signer executed the signs near the center of the torso (e.g., see sign SWIM, phase 3 and 4, Figure 2).

**Graph 4:**
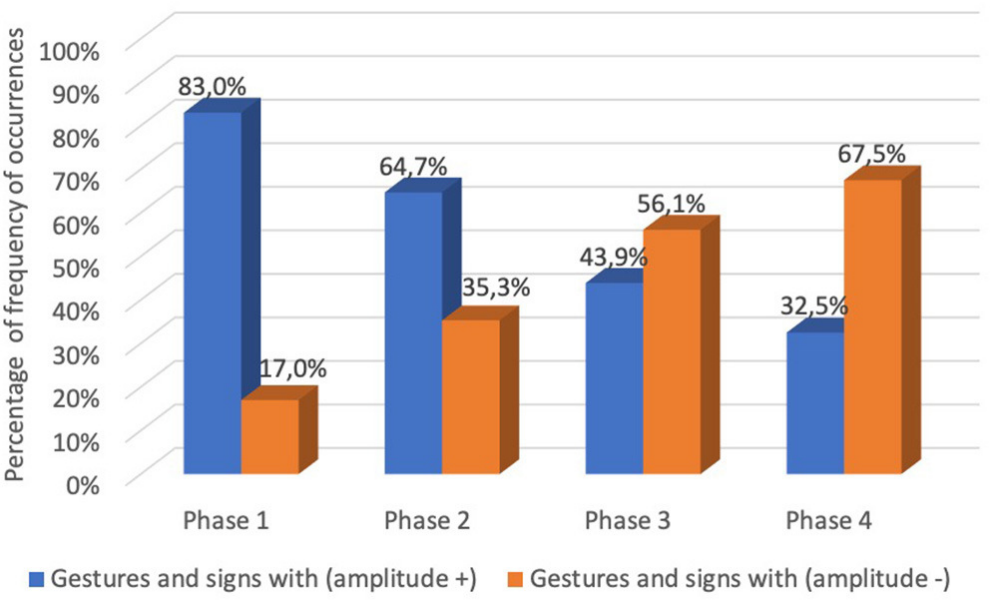
Percentage of frequency of occurrences of gestures or signs with (amplitude +) and (amplitude –) in the analyzed 1,000 gestures and signs along the four phases.

Regarding the amplitude of the occurred gestures and signs, the data revealed that in phase 1, 83% of the gestures and signs occurred with (amplitude +), and 17% with (amplitude –). In phase 2, 64.7% of the gestures and signs occurred with (amplitude +), and 35.3% with (amplitude –). In phase 3, 43.9% of the gestures and signs occurred with (amplitude +) and 56.1% with (amplitude –). In phase 4, 32.5% of the gestures and signs occurred with (amplitude +) and 67.5% with (amplitude –). The data revealed a reduction of amplitude (signing space) along the four phases (Graph 4).

In order to verify the existence of significant differences between each pair of phases considering the amplitude of the 1,000 gestures and signs, the McNemar was applied to the data. The McNemar test revealed a significant difference (*p* < 0.001) between all phases with regard to the amplitude reduction of articulation. Observing the number of gestures and signs that went from (amplitude +) to (amplitude –), it appears that there was a reduction of amplitude (18.3%) from phase 1 to phase 2. From phase 2 to phase 3, the reduction of amplitude was 20.8%. And from phase 3 to phase 4, the reduction was 11.4% (Table 6). The statistical analysis showed that the evolution of pantomimic gestures to signs tended to decrease in the amplitude of the articulations along the four phases.

**Table 6:** McNemar Test: Comparison of pair of phases based on the number of gestures or signs with amplitude (+) and amplitude (–) occurrences considering the analyzed 1,000 gestures and signs.

	**Phase 1 vs. Phase 2**	**Phase 2 vs. Phase 3**	**Phase 3 vs. Phase 4**	**Phase 1 vs. Phase 3**	**Phase 1 vs. Phase 4**	**Phase 2 vs. Phase 4**
**(Amplitude** **+)/(Amplitude** **+)** Nb of gestures and signs with (amplitude +) in both phases	64.7% (647)	43.9% (439)	32.5% (325)	43.9% (439)	32.5% (325)	32.5% (325)
**(Amplitude** –**)/(Amplitude** –**)** Nb of gestures and signs with (amplitude –) in both phases	17.0% (170)	35.3% (353)	56.1% (561)	17.0% (170)	17.0% (170)	35.3% (353)
**(Amplitude** **+)/(Amplitude** –**)** Nb of gestures or signs with (amplitude –) that turned to (amplitude +) from one phase to the next	18.3% (183)	20.8% (208)	11.4% (114)	39.1% (391)	50.5% (505)	32.2% (322)
**(Amplitude** –**)/(Amplitude** **+)** Nb of gestures or signs with (amplitude +) that turned to (amplitude –) from one phase to the next	0% (0)	0% (0)	0% (0)	0% (0)	0% (0)	0% (0)
Total nb of gestures and signs	100% 1,000	100% 1,000	100% 1,000	100% 1,000	100% 1,000	100% 1,000
Teste McNemar	*p < *0.001	*p < *0.001	*p < *0.001	*p < *0.001	*p < *0.001	*p < *0.001

## Discussion

In the present research, the data revealed a transition from pantomime to conventionalized signs within a few months through a concentration of articulation, as found in previous research (Frishberg, 1975; Nyst, 2007).

Sao Tome Sign Language did not emerge *ex-nihilo*. Similarly to creoles, this sign language emerged from the absence of a conventional language model (Adone, 2012). This similarity has been pointed out by several studies (Kegl et al., 1999; Sandler et al., 2005; Adone, 2012), who claimed parallelism between creole languages and young sign languages in structural properties. Sao Tomé Sign Language was naturally born from the contact and interaction between deaf people attending a project in a school, i.e., in sociolinguistic context (Bickerton, 1990; Lane et al., 1996). Our participants came into the project without any previous linguistic models, although the majority of them had some vital home signs to communicate basic needs with their hearing family. Complexity in this new language begun to grow when they began to name objects, feelings, and actions together, based on their previous home signs, and negotiated the best sign for representing the referent shown in the cards. If the first expression of their communication was pantomimic, gradually it decreased, giving space for repeatedly-used signs to name the referents.

Established sign languages have linguistic rules for phonology, morphology syntaxis and semantic structures. Sign languages use hands and arms as primary articulators. Early research on sign languages was most focused on manual articulators: hands, and the configurations, orientation, and movement of hands and arms. Non-manual articulators (Head, torso, face and mouth) during signing have been investigated, recognizing that signers use non-manual articulators in systematic, rule-governed ways in which linguistic content can be conveyed (e.g., Baker-Shenk and Padden, 1978). Non-manual actions are coordinated with movements of the hands during sign production. The head, mouth, torso and legs are agreed to be the non-manual articulators in sign languages.

In a young or newly-born sign language, pantomimic gestures involve engagement of the whole body or parts of whole body as articulators. In our corpus, we identified that the torso, head and legs were present as articulators along the four phases under analysis. Although the use of the analyzed articulators had a tendency to reduce over time, the head was the most prevalent articulator. In future research, it would be interesting to see if and how some of these articulators stabilize in a systematic way to convey linguistic content.

Gestures and signs evolved to use a smaller signing space and became more effortless and economical. This finding is concomitant with previous studies (Lavoie and Villeneuve, 2000; Takkinen, 2003) which have found that signers change from compact signing (e.g., movement of the shoulder) to extended signing (e.g., movement of the elbow). Signers of a new language may become gradually more efficient in the use of articulators reducing the production effort (Dachkovsky et al., 2018). This change is motivated by ease of articulation.

Emerging sign languages show some stabilization and conventionalization in early stages of their development (Sandler et al., 2005; Padden et al., 2013; Mineiro et al., 2017; Dachkovsky et al., 2018). However, they still lack regular and consistent structures as they develop over time and across signers within a signer community. The growth of interaction between the deaf participants starting to form a signer community and the cultural pressure to have a common efficient language to communicate facilitated, in our view, the evolution of pantomime into conventionalized signs.

None of our participants had ever met before, and as they begun to know each other and create social relations and social networks, the language began to develop more and more. We can see differences in all phases of this study, which also reveals the interaction between them. Firstly, the participants were together in a shared space. Secondly, they begun to share and to create commitment alliances in order to have a community and a language built in shared values.

Pantomime played a significant role when our participants met for the first time. As stressed by Arbib (2012, 2013) first pantomimes might be “*ad hoc*” or ingenuous. It was the only way they could speedily establish a connection and begin to communicate, even if the unstandardised character of pantomime leads to the disregard of communication efficiency (Zywiczyński et al., 2018). As time went by the pressure to have a more efficient system, effortless in terms of articulation and quicker to produce, led to the emergence of new signs in which iconicity as a major vehicle of semantic transmission (Frishberg, 1975) was transformed into a lighter version, with participants giving up the whole-body commitment and beginning to use the manual articulators as a preferred source. Using both hands instead of the whole body (see example GOAT in Figure 2) leads to a reduction of amplitude (signing space) and a reduction of the iconicity of the sign. This link between concentration of articulation and the decrease of iconicity was observed in earlier variants of American Sign Language (Frishberg, 1975) and in Adamorobe Sign Language (Nyst, 2007). In the latter a correlation between iconicity and articulatory features, such as manual articulatory preference and signing space reduction, was also observed (Nyst, 2007).

In our data, non-manual articulators such as the torso, legs and head lost their importance in all the phases and signs begun to be articulated with manual articulators, specifically, both hands, with a slight preference for the articulation of the dominant hand. The continuous recruitment of body articulators for linguistic functions can shed some light into archaic communicative capacity and maybe one of the keys in language evolution research regarding core linguistic properties and their origins (Sandler, 2018).

These results can also be found when in a young sign language discourse, the increased complexity is reflected by the conventionalization, convergence, reduction of articulatory effort and changes in number and type of articulators such as the torso or trunk (Dachkovsky et al., 2018). The growth of conventionality identified in our study showed us the advantage of reducing length and complexity in the sign's evolution.

A recent study found that the miniature artificial sign language created in the lab evolved from inefficient gestures into gestures exhibiting systematicity and efficiency through a combination of interaction and transmission conditions (Motamedi et al., 2019).

Another exciting study showed that participants who repeatedly interacted using graphical signs (Pictionary Game) transitioned from iconic motivated signs to arbitrary symbolic ones. This study demonstrates that during early stages, there is more dependence on creative inferential mechanisms related to problem-solving, and that as time goes by the recruitment of these mechanisms slows (Sulik, 2018).

All those investigations corroborate our crucial point. That is, there seems to exist a continuum between pantomime and lexicalized gestures in a new sign language, and it is a question of time of how the system absorbs the rules and begins systematic recombination of segmented signs. In mature sign languages, we can also find a continuum between gestural and linguistic elements as part of linguistic conventionalization (Nyst, 2007). Interaction and transmission lead to activation of increasingly economical cognitive mechanisms for creating the meaning of novel signs during symbolization afforded by shared knowledge. This process seems to be a natural tendency of simplification due to economy of action, as was described in the 70 s by researchers on sign languages (Frishberg, 1975; Klima and Bellugi, 1979; Kendon, 1980, 1988). Symbolization engages a change from complex, iconic signals to simpler ones, lighter in terms of iconicity and leading to arbitrary signs.

In this sense, we claim that both pantomime and conventionalized signs are intertwined with language. At the beginning there is a pattern of action or the representation of an object, performed or described in elaborated movements, which then loses its complexity and is reduced to less complex movements—for instance, “two-handed forms tend to become one-handed” (Kendon, 2004, p. 308)—with a higher level of abstraction. In sign languages studies (Frishberg, 1975; Brentari, 1998; Moita et al., 2018), a manual complexity loss has been observed through the “weak-drop” phenomenon, in which there is a deletion of the unmarked hand in the two-handed symmetric signs throughout the history of the sign language, among the youngest generations of signers and during informal conversation. For spoken language there is a similar conventionalization process, with pantomimic representations at the beginning and the arbitrary vocal signals which have replaced them at the end point (Corballis, 2014). Research claiming the gestural origins of language before vocal language (Corballis, 2003) may find reinforcement if we see pantomime as an initial level for human communication. Thus, languages might be grounded in pantomime independent of their modality, and thus it may be assumed that pantomime is multimodal.

The powerful motivation for the conventionalization of pantomimic forms comes from the fact that pantomimes are creative and interpreted on the spot. This leads to difficulties in replicating forms and standardizing them.

What is crucial in the process of language conventionalization is the way in which the sign is perceived, understood and agreed upon by the language community. In other words, transmission and face-to-face interaction are necessary for the development of language in humans (Kendon, 2004; Dachkovsky et al., 2018; Motamedi et al., 2019).

In this view, our findings also find support from the neurobiological studies which claim that pantomime and language rely on only partially distinct neural systems, and that neural networks for pantomime generation are not identical for the deaf and hearing groups (Emmorey et al., 2011). Our discussion also reports the relation of language and cognition independently of language use, as language emergence operates with the same cognitive mechanisms for symbolization (Sulik, 2018).

This could not happen without the cultural pressure to have a common language in a community, and for us, the beginning of language in humans is intertwined with this necessity for an efficient system to communicate and cooperate with others. We agree that language is not an instinct (Pinker, 1994; Tomasello, 1995). If it was an instinct, homesigns would show us some regularity and conventionalization as well as combinatory and segmental representation, whereas that is not the case, as they have been described to precisely lack exhibition of this (Goldin-Meadow, 2014). Indeed, homesigns displayed by our participants showed a short lexical repertoire, absence of syntactic rules and variability in expressions (Mineiro and Carmo, 2016).

Only with transmission and interaction (Dachkovsky et al., 2018; Motamedi et al., 2019) can language develop in human beings. Our study suggests that sign languages may begin in pantomime and this finding can be a window into language evolution research.

In this study, we aimed to investigate how quickly pantomimic communication turns into lexicalized and conventionalized signs of a newly-born sign language and how this information might contribute to language evolution research. Results displayed in the previous section showed that within 2 years of contact, a community of participants built a shared language. This new language has a lexical repertoire and some linguistic trend rules for phonology, morphology and syntaxis (Mineiro et al., 2017). Our study also clarifies the role of pantomime in a new sign language and how this role is linked with language itself (Armstrong et al., 1995).

In language evolution research, all theories about language genesis are speculative as language leaves no fossils. In this way, all the research has been grounded in topics that fluctuate between vocal, manual or multimodal origin hypotheses (Kendon, 1980, 2004; Corballis, 2003, 2009, 2014; Arbib, 2012, 2013). Our study does not provide answers to this debate as we cannot use data from modern humans who have language-ready brains to answer the question of how language has evolved as human species. Nevertheless, observing an emergent sign language can be a window into disentangling the nature of language and consequently how it might have evolved.

Studies on emerging sign languages (Senghas and Coppola, 2001; Sandler, 2016) provide evidence for how critical properties of the linguistic systems are created. Here, we show how gestures initially produced by participants are unsystematic and resemble pantomime, but come to develop fundamental language-like properties and lead to a gradual increase of regularity and systematic structure. Interaction and transmission are the pillars for the maintenance of communicative efficiency and lead to a gradual increase in regularity and systematic structure.

## Data Availability Statement

The datasets presented in this article are not readily available because the data are with the principal investigator who has written permission to have the video corpus and analyse the data for academic purposes. Requests to access the datasets should be directed to Ana Mineiro, amineiro@ucp.pt.

## Ethics Statement

The studies involving human participants were reviewed and approved by Catholic University of Portugal. Written informed consent to participate in this study was provided by the participants' legal guardian/next of kin. Written informed consent was obtained from the individual(s), and minor(s)' legal guardian/next of kin, for the publication of any potentially identifiable images or data included in this article.

## Author Contributions

AM is the principal investigator, contributed to write the paper, analyse, and discuss the results. IB-M completed the data and analysis, and contributed toward writing the paper. MM contributed to the collection of data, analysis and revision. IG-R and AC-C contributed toward writing and interpreting of the results. All authors contributed to the article and approved the submitted version.

## Conflict of Interest

The authors declare that the research was conducted in the absence of any commercial or financial relationships that could be construed as a potential conflict of interest.
